# The association between self-care and quality of life in hypertensive patients: findings from the Azar cohort study in the North West of Iran

**DOI:** 10.15171/hpp.2018.18

**Published:** 2018-04-18

**Authors:** Maryamalsadat Kazemi Shishavan, Mohammad Asghari Jafarabadi, Nayyereh Aminisani, Mohammad Shahbazi, Mahasti Alizadeh

**Affiliations:** ^1^Department of Community and Family Medicine, Tabriz University of Medical Sciences, Tabriz, Iran; ^2^Road Traffic Injury Research Center, Tabriz University of Medical Sciences, Tabriz, Iran; ^3^Department of Statistics and Epidemiology, Tabriz University of Medical Sciences, Tabriz, Iran; ^4^School of Public Health, Professor Jackson State University, Jackson, Mississippi, USA; ^5^Social Determinants of Health Research Center, Department of Community and Family Medicine, Tabriz University of Medical Sciences, Tabriz, Iran

**Keywords:** Health related quality of life, Hypertension, H-scale, Quality of life, Self-care

## Abstract

**Background:** Hypertension affects the quality of life of patients and their caregivers. The aim of this study was to assess the knowledge and self-care behaviors and health-related quality of life (HRQOL) among hypertensive people.

** Methods:** All people aged 35 years and older with hypertension were invited to participate in this study. Information on self-care behavior for hypertension (H-scale), and health-related quality of life (WHOHRQOL-BRFF) were completed by trained interviewer. Data analysis was done using SPSS 16.

**Results:** The median age of hypertensive patients was 62.5(25th to 75th percentile: 55 to 72 years), the correlation between quality of life and overall self-care scores was not significant(r =-0.048, P =0.520). Physical activity was the only significant predictor for quality of life,showing that the quality of life of hypertensive people increased by 3.371 units per day of being physically active in the cohort study (β =0.223, P<0.01). The only significant predictor of quality of life among the elderly was medication use (β =-0.572, P<0.001). Quality of life of participants decreased 3.456 units per day as a result of medication adherence.

** Conclusion:** No association was observed between self-care and HRQOL total score in hypertensive patients in the study. Among the self-care domains, only medication adherence and physical activity had significant association with social health. There was a reverse association between smoking and HRQOL.

## Introduction

High blood pressure is a chronic disease that affects the quality of life of patients and their caregivers.^1^ In numerous studies, the prevalence of high blood pressure has been reported between 23% and 50% in Iran.^2,3^ This high prevalence requires the allocation of resources as well as prioritizing patients and their lifestyle conditions. Clinicians and policy-makers use health-related quality of life (HRQOL) measurements as a tool for disease management and health policy making.^4^

HRQOL is a measure consisting 6 domains assessing physical and psychological health, level of independence, social relationships, environment in which people live and spiritual, personal and religious believes.^5^ This concept shows how individuals relate to the community and culture they live in and direct their goals. It shows their attitude to the position they have in life as well.^6^

HRQOL in patients with hypertension depends on various factors such as age, sex, dyslipidemia, body mass index (BMI), glucose intolerance, smoking, and terminal organ damage such as kidney disease and retinopathy.^7^ These factors have proven effects in many patients. A few studies have been done on quality of life and self-care behavior. Studies have shown that HRQOL in patients with hypertension can be influenced by the patients’ knowledge and beliefs about disease, measurement and control of blood pressure, and how to take medications.^8^

Self-care is defined as any action taken to maintain one’s health and prevent disease, including health, nutrition, and lifestyle, environmental conditions, income and socio-economic status and self-treatment.^9^ Self-care in patients with high blood pressure has been announced as a key step in reducing hypertension pandemic.^10-12^ Self-care behavior is considered a major determinant of blood pressure control in some studies. In other studies, behaviors such as proper use of medications, exercise, proper nutrition, and weight control are further examples of self-care. However, factors other than age, sex, marital status, employment, duration of hypertension, knowledge and beliefs about it and self-efficacy related to self-care and hypertension have been emphasized.^11^ Some studies have examined the impact of chronic diseases on HRQOL. It has been reported that people with hypertension have lower quality of life compared to those with normal blood pressure.^11-13^

Studies such as TOHP (Trials of Hypertension Prevention)^14^ and TOMHS (Treatment of Mild Hypertension)^15^ demonstrated that quality of life can be improved by reducing blood pressure. The use of medication as first line alone, however, was not effective on reaching target blood pressure in these patients in large part because many patients do not take medications as prescribed. Some meta-analyses recommend lifestyle changes to lower blood pressure, including activities such as a diet for weight loss, regular exercise, and a reduction in salt and alcohol intake for people with hypertension.^15^ A tool for measuring self-care was created and validated based on these recommendations.^12,16^

Some studies have been conducted on self-care and HRQOL in the elderly (over 65 years). It has been seen in diseases such as heart failure and the quality of life associated with self-care in different dimensions. However, limited information is available for the middle age group.^17^ Some studies have done on the relationship of these two factors in type 2 diabetes mellitus, but no clear relationship has been reported.^15^

Given the contradictory results of other studies, especially in the areas hypertension and the need for planning and health policies for the target population, we conducted this study to assess the association between self-care behaviors along with therapeutic interventions and HRQOL among hypertensive people in the pilot phase of the Azar cohort study; a state-level of a nationwide cohort study entitled “PERSIAN Cohort Study”^18^ in Iran (http://persiancohort.com/) in order to initiate educational programs for hypertension control based on self-care interventions.

## Materials and Methods

### Introducing Azar cohort study

The present study was conducted based on the pilot phase of the Azar cohort study. The Azar cohort study, a state-level of a nationwide cohort study entitled “PERSIAN Cohort Study”^18^ in Iran that was launched in 2014 in different geographical regions of Iran, mainly aimed at assessing a comprehensive range of different biomarkers, lifestyle, socioeconomic factors, and health-related factors of common non-communicable diseases among Iranian adults. The Azar cohort study has been conducted by Tabriz University of Medical Sciences in Shabestar, a county located in East Azerbaijan province. All adults from 35 to 70 years of age were invited to take part in this study if they met the inclusion criteria (permanent resident of the city, ability to respond to the questions, Iranian origin). Up to 20 000 people will eventually be recruited in this study, which will be sufficient sample for the main outcomes of interest such as cardiovascular disease, diabetes, etc. The pilot phase of the Azar cohort study was conducted in Khameneh, one of the cities in Shabestar County, between October 2014 and Jan 2015.

Regarding the purpose of the pilot study, all residents of Khameneh who were 35 years and older were invited by phone to participate in this cohort study. Eligible people were invited to visit the Azar cohort center for the assessment. Information related to demographics, socioeconomic status, lifestyle factors, diet, and medical history of diseases was collected via questionnaire by trained interviewers. General physical examination was conducted by a general physician. Blood pressure (BP) and anthropometric measures such as weight, height, waist and hip and wrist circumference were measured. Hair, nail and urine samples were collected from participants. BP was measured by a trained nurse/midwife using a Richter sphygmomanometer (Germany). BP was measured after the participant had rested in a seated position for 15 minutes, and was recorded twice, at approximately 10-minute intervals for each arm.

Study participants were classified into 3 groups based on their BP: normal BP (SBP <120 mm Hg and DBP < 80 mm Hg), pre-hypertensive (SBP ≥120 mm Hg but <140 mm Hg or DBP ≥80 mm Hg but <90 mm Hg), and hypertensive (SBP ≥140 mm Hg and/or DBP ≥90 mm Hg and/or self-reported use of antihypertensive medication).

### Participants

Of 1038 participants in the pilot phase of Azar cohort, 952 were less than 70 years of age, and 86 were over 70 years old, of these, 181 were non-elderly with hypertension and 47 were hypertensive elderly participants. Basic information of the elderly participants was not included in the PERSIAN cohort study but some of their information was accessible in the files. All people with hypertension were invited to Khameneh where the Azar cohort study was being conducted to complete self-care for hypertension (H-scale questionnaire) and the World Health Organization Quality of Life Questionnaire (WHOHRQOL-BREF). While providing health care for all hypertensive participants, the questionnaires were completed. Of the 47 elderly participants with high BP, 36 patients (76.5%) and of the 181 non-elderly participants with high BP, 145 patients (80.1%) responded to the invitation and both questionnaires were completed by trained interviewer for a total of 181 patients (elderly and non-elderly),73 males (40.3%) and 108 females (59.7%) with high BP.

The interviewer was one of the Khameneh former health worker who was trained for three sessions. The training included how to create rapport, permission and consent forms completion, how to ask questions not to have an inductive aspect, and how to answer if any possible question was asked by participants. All participants completed an informed consent before answering any questions.

### Instruments

The WHOHRQOL-BREF was extracted from the original questionnaire of 100 question from 1996 and its validity and reliability was examined for use in Iran. Cronbach’s alpha for physical health, psychological health, social and environmental health were 0.72, 0.70, 0.52, 0.72 respectively.^19^

The hypertension self-care activity levels (H-SCALE) is an instrument to assess self-care in hypertensive patients.^12^ It includes items assessing medication adherence (3 items), practice of weight management activities (10 items), physical activity (2 items), smoking exposure (2 items) alcohol intake (2 items) and eating healthy, defined as low fat and low salt diet (12 items).^11,12^

The questionnaire was translated into Persian using the standardized method of forward-backward: The English questionnaire was translated into Persian by one of researchers and then discussed by eight experts (community medicine specialists, epidemiologists) and approved. After that the approved Persian version was translated into English by a person fluent in English without any knowledge about the original text. The second English version was sent to a native English speaker who was asked to compare with the original version and specify the contradiction. Finally, after being approved, the final version was translated into Persian and approved by those experts.

The final version was completed by 30 patients. “Medication adherence “subscale consisted of 3 items (α = 0.7), “eating healthy” subscale consisted of 12 items (α = 0.75), “smoking exposure” subscale consisted of 2 items (α = 0.78) “physical activity” subscale consisted of 2 items (α = 0.80), in “Weight management” subscale consisted of 10 items (α = 0.78) and “alcohol intake” subscale consisted of 3 items (α = 0.70).

### Statistical analyses

Data analysis was done using SPSS 16 (SPSS Inc., IL, Chicago, USA). The calculated frequency and central tendency and dispersion were used for descriptive analysis. Pearson correlation coefficient was used to determine the correlation between quality of life and self-care scores. An independent *t* test was used to compare the average score of quality of life in the elderly and non-elderly. A one-way ANOVA was done to compare the occupation relationship (housewives, workers, employee, retired) with quality of life. Also, multiple linear regression analysis was done to predict quality of life based on self-care areas in total and in elderly sub groups. In all analyses *P* < 0.05 was considered statistically significant.

## Results

Of 47 hypertensive patients above 70 years of age and of 181 hypertensive patients under 70 years of age, 36 and 145 individuals participated for the interview of quality of life and self-care respectively.

The median age of hypertensive patients was 62.5 (25th to 75th percentile: 55 to 72 years), the minimum age was 36 and maximum was 88 years. In terms of gender distribution, 40.3% were males and 59.7% were females. Descriptive findings are summarized in Table 1.

In the descriptive study of self-care, only physical activity had low scores compared to other lifestyle-related activities. Among elderly group, 20 (55.6%) patients had 30 minutes of physical activity (except for the routine activities) and 50 (34.5%) non-elderly had no physical activity during the week before the interview. In total, 20 males (27.4%) and 50 females (46.3%) had no physical activity, there was a significant difference in the average of physical activity between men and women (mean difference = 1.99, 95% CI: ‌0.98-3.01).

The correlation coefficient of -0.048 was obtained between the scores of quality of life and self-care. This correlation coefficient shows an inverse relationship with weak strength between the self-care and quality of life scores but is statistically not significant (*P* = 0.52)

A multiple linear regression analysis was done to predict the quality of life based on self-care. Since none of the participants reported alcohol intake, alcohol intake was not entered the model. A significant relation was found as follows: F (5,177) = 3.673, *P* < 0.005 with R^2^ = 0.094.‌ Taking into account all predictors simultaneously, the medication adherence, eating healthy, physical activity, smoking exposure and practice of weight management activities, account for 9.4% variance in health related quality of life. Medication adherence contributed for 2.6% of this total variance, eating healthy, physical activity, smoking exposure, account for 0.4%, 4.6%,1.8% respectively and weight management activities accounts for non. The quality of life of hypertensive patients was predicted by the model; Physical activity explained a significant amount of unique variance in health related quality of life showing that the quality of life of hypertensive people increased by 3.371 units per day physical activity in the study (Table 2)

**Figure 1:**
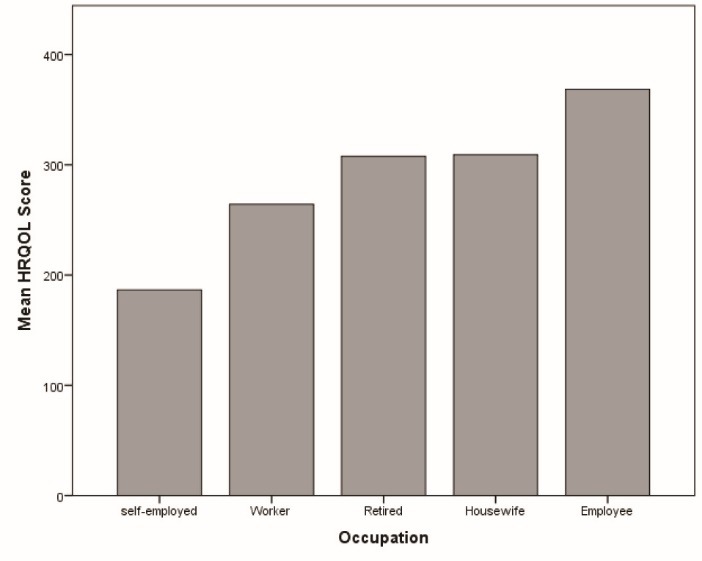
Mean scores of health related quality of life in hypertensive women in the Azar cohort study.

A one-way ANOVA was done to compare the occupation relationship (housewives, workers, employee, retired) with quality of life. There was a statistically significant difference between health related quality of life in different occupation groups in women as determined by one-way ANOVA (F (4, 83) = 6.2, *P* < 0.001). Mean scores of HRQOL in women with different occupations is presented in Figure 1.

A Tukey post hoc test revealed only being self-employed has statistically significantly lower health related quality of life than other occupations, there was no statistically significant difference between health related quality of life score in men with different occupations. (*P* = 0.665)

The score of the quality of life of the elderly was significantly less (*P* = 0.006). An independent *t* test was also used to compare the quality of life in men and women. There was no significant difference in the quality of life score (*P* < 0.001).

A significant regression equation was calculated: F (5,30) = 4.300, *P* < 0.005, with R² = 0.417 Taking as a set, the predictors medication adherence, eating healthy, physical activity, smoking exposure and practice of weight management activities, account for 41.7% variance in health related quality of life. In this model, contribution of each predictor in total variance in HRQOL comes as follows: Medication adherence contributed for 19.8% of this total variance, smoking exposure, physical activity, weight management activities, eating healthy account for 12.5%, 5.4%, 3.9%, 0.1%, respectively.

The significant predictors of quality of life among the elderly was medication adherence and smoking exposure. Quality of life of hypertensive people decreased 3.456 units per day due to being adherent to medication, holding other variables constant. In addition, a statistically significant association was observed between smoking and quality of life. The quality of life decreased in patients with high BP by increasing smoking days of exposure (*P* = 0.015) (Table 3).

No significant regression equation was found to predict the quality of life in the sex subgroup or non-elderly participants

Self-care areas (medication adherence, physical activity, smoking exposure, weight management activities, healthy eating), even after controlling for age, sex, education and marital status, and employment, had no significant relationship with quality of life. (*P* > 0.0.05).

Among the self-care areas, medication adherence had a significant association only with social health (social support, interpersonal relations) (*P* = 0.014), while it had no significant association with physical health and general health. (*P* = 0.329, *P* = 0.456, respectively). Physical activity had a significant association with social health (interpersonal communication and social support) (*P* = 0.009) but no statistically significant association with physical and mental health (*P* = 0.093 and *P* = 0.736, respectively).

Weight management had no significant association with social health but had a significant relationship with a healthy environment (financial security, environment safety) (respectively, *P* = 0.15, *P* = 0.023).

Based on the one-way ANOVA test, education had no significant relationship with quality of life score. (F (6,138) = 1.66, *P* = 0.134).

## Discussion

This study aimed to examine the association between self-care behaviour and quality of life in group of people with hypertension. The results showed no significant linear correlation between self care and health related quality of life; however, in the multivariate regression test, a significant regression equation was found between the two, that is the self-care can affect quality of life in general, but there is no linear relationship between the two.

In most previous studies, a significant relationship has been reported between self-care and BP control in which a self-care relationship was observed on systolic and diastolic BP reduction (intervention studies).^15,20-26^ There are limited studies about the relationship of self-care with quality of life, which is an principal outcome of hypertension control; however, the impact of hypertension on quality of life has been reported by Cappuccio et al, so that these patients have a lower quality of life, mostly due to the treatment of hypertension rather than the disease itself.^26^

Wang et al showed that hypertension and its complications reduced the score of quality of life.^13^ The relationship between different areas of self-care and quality of life, including physical health, mental health, social health, environmental health, and general health was examined and the results showed that among the self-care areas, only medication adherence had a significant relationship with social health (social support, interpersonal relations).

Physical activity had a significant relationship with social health but no relationship with physical and mental health. Shinagawa et al examined mental health and BP control as BP was monitored by patients, and indicated a relation between BP control and depression which was not shown in our study.^27^ In a quasi-experimental study conducted by Agajani et al on the relationship of self-care education with quality of life, it was shown that self-care education impacted all aspects of quality of life; however, the impact on the physical health aspect of quality of life was not significant, but this intervention showed a significant relationship in other aspects. It should be noted that the Agajani et al study was an interventional study measuring the effect of self-care education on quality of life.^28^

One of the most important aspects of self-care is adherence with a regular prescribed medication regimen; in the present study, a statistically significant relationship was found between this domain of self-care and social health domain on the quality of life. But there was no statistically significant relationship with physical health and general health.

Given that this is a cross-sectional study, a cause-effect relationship between these cannot be established; that is, social health improves medication adherence or vice versa is neither accepted nor rejected in this study.

A study was conducted by Hanus et al in which a direct relationship between regular, compliant use of prescribed medications and quality of life has been reported. Patients who are regularly compliant with a prescribed medication regimen had higher scores in mental and social areas, which indicates the impact of mental health on medication adherence.^29^ The lack of relationship between medication adherence and mental health in our study and contradictive results with the Hanus study were due to differences in cultural conditions between the two populations studied.

Since there was no linear relationship between HRQOL and self-care in this study, it can be concluded that other determinants affect the HRQOL and the quality of life improvement in hypertensive patients by only self-care cannot be expected. So that in a study, Adedapo et al examined determinants of HRQOL in patients with hypertension and showed that high BP and medications could reduce quality of life. Factors other than self-care, such as income and socioeconomic level have a significant impact on HRQOL.^8^

Of behaviors constituting various aspects of self-care, participants with more physical activity had better quality of life. Women and elderly had less physical activity in this population that could be related to cultural and environmental conditions that make physical activity difficult or inaccessible, especially in women. It is also possible that there is not enough education and training regarding the importance of physical activity in the elderly. Most elderly people suffer from bone and joint pain and various health problems, who may render physical activity unsuitable. The other explanation is; old people have less physical activity due to their perception about being ill.

In the present study, there was a significant difference in the quality of life between men and women with hypertension: the quality of life for men was better than women. There was no relationship between having a job and quality of life in men, but among women, employees had better quality o life. This could be due to regular revenue and perceived job security

In this regard, many studies have been done on hypertension and job as well as quality of life. In a recent study conducted at Stanford University, the relationship between workplace and psychological and social factors on the prevalence of hypertension was shown.^30-33^

Another important point in this study was an inverse relationship between medication adherence and quality of life in the elderly. The low quality of life in the elderly is one of the findings of this study, which was found to be far lower even in those who adhere to a regular medication schedule. This could be due to social, cultural, and family reasons including social networks, social capital or low family support. In spite of regular medication regiment compliance, various aspects of physical and mental, social and environmental quality of life are low in the elderly.

Compliance with a medication regiment is one dimension of self-care. Its impact on quality of life in people under 65 years was related only with social health. This relationship suggests that interpersonal relationships and social support play roles in the medication regiment compliance at any age. Several studies have been conducted about medication compliance and acceptance of treatment recommendations in hypertension and its determinants. One of the latest studies in this field was the study of Meinema et al in the Netherlands in 2015, showing that people who were more compliant with prescribed medication regiments received more support from family compared to those who did not.^30^ Another study, a meta-analysis, showed relationship between treatment acceptance and social support.^34^ The impact of social support and family quality of life of hypertensive patients and their treatment acceptance have been emphasized in other studies.^34^ Another notable result in this study was the relationship between “smoking” as a self-care dimension with a lower quality of life. Quality of life score in people who were smoking was significantly low.

In a recent study conducted in Australia, it was shown that physical activity in people with high BP and other metabolic syndrome aspects tended to be lower. People who were physically active reported better quality of life in physical aspect. This result is consistent with results obtained in the present study and other similar studies. The percentage of non-smokers with and without hypertension and other aspects of metabolic syndrome was not different, but non-smokers had higher scores in the mental aspect of quality of life, which was similar to the results of our study. The low quality of life in smokers could be for many reasons, including the underlying psycho-social causes of smoking.^35^

A comprehensive cross-sectional study conducted in China in 2016 in which 1224 hypertensive elderly were studied and the quality of life and its relationship to lifestyle and other factors were examined. In patients with hypertension, the scores of physical and public and social health were generally lower. The level of education and income, smoking and family relationships were associated with quality of life. So that hypertensive people with a higher education, married, and good income had a better relationship with family and higher quality of life score was reported. The results of this study are consistent with the current study in terms of dimensions and social determinants such as income, social and family relationships. Unemployed participants had lower scores in the social and environmental aspects in their quality of life compared to the physical and psychological aspects. The result of this study were similar to ours in terms of smoking. However, in this study, the effect of smoking was shown on the physical and physical pain aspects of quality of life.^36^

## Conclusion

This cross-sectional study was conducted to determine the causes of self-care situations and their impact on quality of life. Since it was done in the pilot phase of the Azar cohort study, it was not possible to do a longitudinal study, or to study questions on effect of intervention. The sample size was small compared to other studies, but in this study, all patients diagnosed of hypertension in the pilot phase of cohort Azar study were enrolled.

Policy recommendations include greater emphasis on behavioral and socio-economic aspects in patients with hypertension. Self-care as a component of personal care cannot merely be effective in improving the quality of life on people with hypertension and the social determinants; cultural interventions for improving psychological conditions of women and housewives compared with men and enhancing social activities for the elderly and the retired should be noted along with it. Although measuring quality of life using tools such as quality of life questionnaires in a quantitative study is inevitable, different aspects of life for everyone has different effects on perceptions of their quality of life. We recommend that models and items be predicted in using such tools to calculate the final score of quality of life to proper weighing the different aspects of quality of life by any participant individually, then the final score will be a more accurate estimation of the quality of life of that individual.

It is also recommended that further intervention studies be done and actions such as self-care and self-management and participatory decision-making be compared and the best intervention be determined.

## Ethical approval

The project was approved by Ethics Committee of Tabriz University of Medical Sciences in 2016 (The ethical code: TBZMED.REC.1394.1206).

## Competing interests

Authors declare no conflict of interest.

## Authors’ contributions

MSH and MA encouraged MK to investigate the association of health related quality of life and self care in hypertensive patients and supervised the findings of the research. MK and MA and N.A carried out the study. MK and MA wrote the manuscript with support from MAJ and MSH and NA. MAJ verified the analytical methods. All authors discussed and contributed to the final manuscript.

## Acknowledgments

The researchers would greatly appreciate Tabriz Health Services Management Research Center for providing us with the financial resources and grants for doing this project. We would also like to render our thanks to the hard working personnel of Khameneh Health Center and Khameneh citizens for their cooperation.

**Table 1:** Frequency table/ of education, occupation and marital status of participants (35-70 years old)

	**Men** **No. (%)**	**Women No. (%)**	**Total No. (%)**
Education			
Illiterate /5 years of primary education (Elementary school)	19 (32.2)	39 (45.8)	58 (40.2)
8 years of primary education (secondary school)	12 (20.3)	12 (14.0)	24 (16.6)
12 years of primary education (diploma)	12 (20.3)	18 (20.9)	30 (20.7)
Associate degree	5 (8.5)	3 (3.5)	8 (5.5)
Bachelor or higher degrees	7 (11.9)	2 (2.3)	9 (6.2)
Unknown	4 (6.8)	11 (12.8)	15 (10.3)
Total	59 (100)	85 (98.8)	144 (99.3)
Occupation			
Housewife	0 (0)	71 (82.6)	71 (49.0)
Employee	6 (10.2)	3 (3.5)	9 (6.2)
Retired	12 (20.3)	7 (8.1)	19 (13.1)
Worker	21 (35.6)	2 (2.3)	23 (15.9)
Self-employed	20 (33.9)	2 (2.3)	22 (15.2)
Total	59 (100.0)	85 (98.8)	144 (99.3)

**Table 2:** The relationship between quality of life and self-care dimensions based on linear regression analysis (n = 181)

Predictors*	**B (95%** **CI)**	**Beta**	***P*** ** value**	**R** ^ 2 ^
Medication adherence	-0.865 (-1.78, 0.54)	-0.136	0.065	2.6%
Healthy eating	-0.556 (-1.68, 0.576)	-0.070	0.334	0.4%
Physical activity	3.371 (1.182, 5.559)	0.223	0.003	4.6%
Smoking	3.416 (-0.214, 7.046)	0.133	0.065	1.8%
Weight management	-0.110 (-1.564-1.343)	-0.011	0.881	0%

*Dependent variable: health related quality of life total score, R^2^ = 0.094, *P* value < 0.05 was considered significant.

**Table 3:** Relationship between quality of life and self-care dimensions based on linear regression analysis in the subgroup of the elderly (n = 36)

Predictors*	**B (95%** **CI)**	**Beta**	***P*** ** value**	**R** ^ 2 ^
Medication adherence	-3.456 (-5.329, -1.583)	-0.572	0.001	19.8%
Healthy eating	0.543 (-2.629, 3.716)	0.051	0.729	0.1%
Physical activity	3.397 (-1.294, 8.487)	0.215	0.144	5.4%
Smoking	-131.966 (-236.032, -27.900)	-0.384	0.015	12.5%
Weight management	-2.392 (-5.836, 1.051)	-0.206	0.166	3.9%

*Dependent variable: health related quality of life total score, R^2^ = 0.417, *P* value < 0.05 was considered significant.
